# Book review: Basics in medical education

**DOI:** 10.3352/jeehp.2011.8.8

**Published:** 2011-07-30

**Authors:** P. Ravi Shankar

**Affiliations:** Department of Medical Education, KIST Medical College, Lalitpur, Nepal.

The Flexner report and Abraham Flexner occupy a dominant place in modern western medical education. Flexner suggested the traditional basic sciences and clinical sciences division of medical education and emphasized the 'science' of medicine. It may surprise many of you to know that Flexner was not a medical educator and not even a medical doctor. He was a headmaster of a little known school in the United States. This is one of the interesting facts I discovered in the book titled 'Basics in medical education' by two medical educators from the National University of Singapore. The Flexnerian model has a number of deficiencies. I was pleased to be reminded about the 'role' of basic science departments opening the floodgates of knowledge convinced their subject is the most important in medicine. Reforms have concentrated on integrated learning of the basic science subjects and underlining their importance in the practice of medicine. I was specially intrigued by the colonial influence on medical education in Asia. Nepal initially developed a different system of education with inputs from different regions. However with opening of private schools and recruitment of a large number of faculties from India the Indian and the old British influence has begun to be felt if not in theory at least in practice.

The book has been divided into ten sections starting from 'A bird's eye view of medical education' and ending with 'Evaluation of impact.' The other sections are 'Educational concepts and philosophies,' 'Defining outcomes and objectives.' Instructional methodologies for general and clinical teaching and for problem-based learning and assessment are also covered. Educational philosophies are not easily understood by many educators and the authors have explained in a simple manner the difference between the instructivist and the constructivist philosophy of education. Constructivism is the basis of modern education philosophy and encourages self-learning and prepares learners for life long learning. In South Asia learning styles of students are influenced by the system of assessment which stresses rote learning and reproduction of information. I had previously studied learning styles among students and found most use a strategic learning style using both deep and surface learning according to requirements. In our institution we conduct regular teacher's training workshops and I conduct the session on adult learning which is based on constructivism and self-learning philosophies. The authors define a curriculum and mention that the first step is to identify the institution's mission and its objectives. I feel this step is often not done in South Asia where the curriculum is handed down by the medical council or the university. The problem is the same university has a diversity of medical schools with different stakeholders and objectives. Having been involved in innovations in medical education the section on strategies for implementation of educational innovations was of special interest. Educational objectives are important and we start our training workshops with a session on writing learning objectives. The three domains of Bloom's taxonomy have been well described by the authors. The affective domain receives less emphasis in traditional curricula and modern innovations like learning medical humanities emphasize this neglected domain. The list of action verbs which can be used while framing objectives will be very useful to the teacher.

Lectures continue to be a dominant method of teaching due to large class sizes and other reasons and the chapter on making lectures more effective will be helpful. Periodic pause and review, carefully crafted questions and answers, immediate test and activities are mentioned. We have been using some of these methods in our lectures. I have been using small group activity-based learning in pharmacology practical sessions and in the Medical Humanities module and had learned about this during my fellowship at Foundation for Advancement of International Medical Education and Research. The chapter on understanding small groups presented the needed information in a simple and effective manner. Role plays are very effective in learning communication and counseling skills and we have been using them for a long time in both Pharmacology and the Medical humanities. Role plays do not need expensive resources and can be conducted easily in resource challenged settings.

The chapter on questions and questioning techniques is within the specific focus of this journal and is a very important area in medical education. Assessment drives learning and it is unfortunate often teachers are unable to modify assessment methods which are decided by universities and other agencies. Clinical bedside teaching has a number of built in advantages. It takes place in a patient setting, is conducted in small groups, is activity based, concentrates on solving a patient problem and the clinical importance of the subject matter is underscored. The authors describe objective structured clinical examination and norm and criterion based assessment. Problem-based learning (PBL) has generated debate in medical education and many schools follow at least a partially problem-based curriculum. PBL is active, uses clinical problems for learning and is in consonance with adult learning principles. The authors give an excellent overview of PBL. The methods of assessment of PBL were of special interest to me and the authors have described it in a lucid and simple manner.

In South Asia the process of evaluation and assessment has traditionally concentrated on students and the benefits of the process for teachers and faculty administrators among others has not been considered. Formative assessment (during the course of study) and summative assessment (for certification purposes at the end of the course) has been well described. We do not commonly subject our assessment instruments to rigorous scrutiny. Assessment is receiving belated emphasis and the quality of assessment instruments is being rigorously evaluated. Multiple choice questions (MCQs) are commonly used and constructing a good MCQ and questions assessing different set of skills is an essential prerequisite for a teacher. The methods of assessing quality of MCQs will be useful. A number of modifications to essay questions have been done to make them more objective and reduce bias in marking. Oral examinations or viva-voce has come in for a lot of criticism recently and modifications have been carried out to reduce inter examiner variability. Recent advancements in assessment like standardized patients and portfolios are also covered. Research in medical education has been briefly described and the list of resources for readers and glossary of terms will be helpful.

Each chapter ends with a brief summary and a guide to further reading. Simple and lucid language is a feature of the book and the authors have described even difficult terms using simple examples. The book will serve as a good first introduction to medical school faculty to the subject of medical education. Different important aspects like teaching, assessment and recent innovations have been well described. I would strongly recommend this book to readers.

